# In-Stent Restenosis Progression in Human Superficial Femoral Arteries: Dynamics of Lumen Remodeling and Impact of Local Hemodynamics

**DOI:** 10.1007/s10439-021-02776-1

**Published:** 2021-04-29

**Authors:** Monika Colombo, Yong He, Anna Corti, Diego Gallo, Federica Ninno, Stefano Casarin, Jared M Rozowsky, Francesco Migliavacca, Scott Berceli, Claudio Chiastra

**Affiliations:** 1 Laboratory of Biological Structure Mechanics (LaBS), Department of Chemistry, Materials and Chemical Engineering “Giulio Natta”, Politecnico di Milano, Milan, Italy; 2 Department of Surgery, University of Florida, Gainesville, FL USA; 3 PoliToBIOMed Lab, Department of Mechanical and Aerospace Engineering, Politecnico di Torino, Turin, Italy; 4 Department of Medical Physics and Biomedical Engineering, University College of London, London, UK; 5 Department of Surgery, Houston Methodist Hospital, Houston, TX USA; 6 Center for Computational Surgery, Houston Methodist Research Institute, Houston, TX USA; 7 Houston Methodist Academic Institute, Houston, TX USA; 8 Malcom Randall VAMC, Gainesville, FL USA

**Keywords:** In-stent restenosis, Vascular remodeling, Peripheral artery disease, Stent overlapping, Longitudinal study, Patient-specific computer modeling, Computational fluid dynamics, Wall shear stress

## Abstract

In-stent restenosis (ISR) represents a major drawback of stented superficial femoral arteries (SFAs). Motivated by the high incidence and limited knowledge of ISR onset and development in human SFAs, this study aims to (i) analyze the lumen remodeling trajectory over 1-year follow-up period in human stented SFAs and (ii) investigate the impact of altered hemodynamics on ISR initiation and progression. Ten SFA lesions were reconstructed at four follow-ups from computed tomography to quantify the lumen area change occurring within 1-year post-intervention. Patient-specific computational fluid dynamics simulations were performed at each follow-up to relate wall shear stress (WSS) based descriptors with lumen remodeling. The largest lumen remodeling was found in the first post-operative month, with slight regional-specific differences (larger inward remodeling in the fringe segments, *p* < 0.05). Focal re-narrowing frequently occurred after 6 months. Slight differences in the lumen area change emerged between long and short stents, and between segments upstream and downstream from stent overlapping portions, at specific time intervals. Abnormal patterns of multidirectional WSS were associated with lumen remodeling within 1-year post-intervention. This longitudinal study gave important insights into the dynamics of ISR and the impact of hemodynamics on ISR progression in human SFAs.

**Supplementary Information:**

The online version of this article contains supplementary material available at (10.1007/s10439-021-02776-1).

## Introduction

Superficial femoral arteries (SFAs) are atherosclerosis prone.^21^ Among the percutaneous approaches to treat atherosclerotic SFAs, the implantation of self-expanding stents is one of the preferred solutions.^26^ However, in-stent restenosis (ISR), caused by excessive neointima growth and unfavorable inward remodeling, represents a major drawback.^32^ The incidence of ISR in this vascular region ranges from 15% to 32%, with a peak between 9 and 15 months after intervention.^19^ Besides the common clinical promoters, such as diabetes and age,^14^ some conditions, including vessel tortuosity,^22^ device length,^14^ stent overlapping,^34^ and biomechanical factors have been identified as concurrent drivers of ISR. Specifically, two biomechanical factors are deemed to promote ISR: (i) the arterial wall injury provoked by the endovascular procedure,^31^ which contributes to ISR initiation, and (ii) the altered local hemodynamics immediately after stenting and during the entire post-operative period,^27^ stimulating both ISR initiation and progression.

Despite its clinical relevance, the process of ISR initiation and progression in human lower limb arteries is still not fully understood.^33^ From a hemodynamic viewpoint, over the last few decades, computational fluid dynamics (CFD) simulations have enabled the local quantification of the hemodynamic forces acting on the luminal wall. Recent studies^8^,^13^ on human femoropopliteal arteries linked the altered local hemodynamics immediately after endovascular procedure with post-operative lumen morphological changes, demonstrating that the wall shear stress (WSS)-based hemodynamic descriptors are moderate but significant predictors of lumen remodeling. However, an investigation of the impact of local hemodynamics on ISR progression and the lumen remodeling trajectory over time was not conducted and could help identify the phase (early or late) of major neointimal formation.

On this basis, the present work focuses on the relationship between local hemodynamics and development of ISR in human stented SFAs during the first year after stent implantation. In detail, a longitudinal study was performed to (i) analyze the lumen remodeling trajectory over time by reconstructing patient-specific SFA models at multiple follow-ups from computed tomography (CT), and (ii) investigate the impact of altered hemodynamics on ISR initiation and progression by carrying out CFD simulations for each vessel and follow-up under consideration. Furthermore, the impacts of the stent length and presence of stent overlapping on the remodeling trajectory and hemodynamics were investigated.

## Materials and Methods

**Figure 1:**
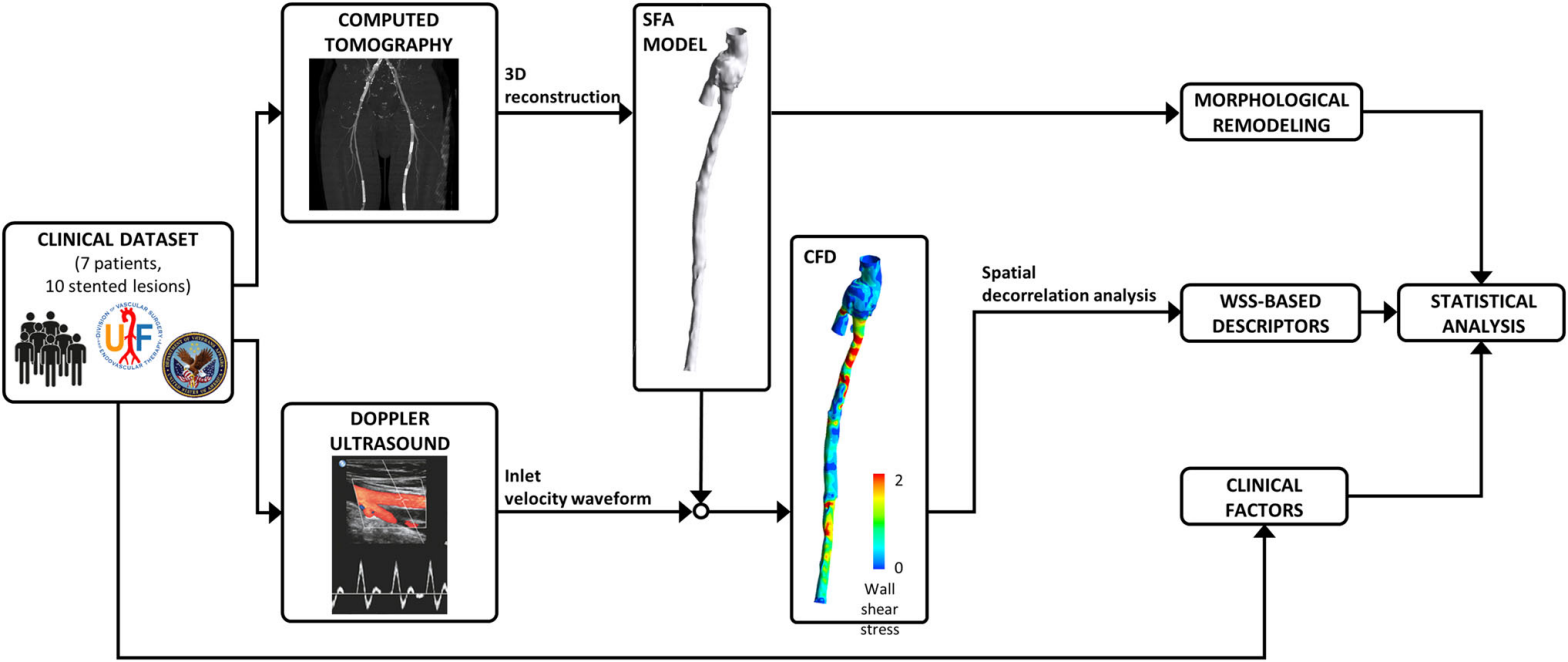
Schematic overview of the workflow.

Figure 1 shows the workflow of the study. Briefly, starting from CT and Doppler ultrasound (DUS) data of human stented femoral arteries, three-dimensional (3D) patient-specific geometrical models of SFAs were reconstructed at multiple follow-ups and used to perform CFD simulations. A morphological analysis quantified the lumen remodeling over time in terms of lumen area change between consecutive follow-ups. Hemodynamics was investigated in terms of WSS-based descriptors. After a preliminary spatial decorrelation analysis, the hemodynamic results were combined with the morphological data to investigate the link between hemodynamics and ISR development. Finally, clinical information, such as age, diabetes, stent length and presence of stent overlapping were included in the statistical analysis.

### Clinical Dataset

**Figure 2:**
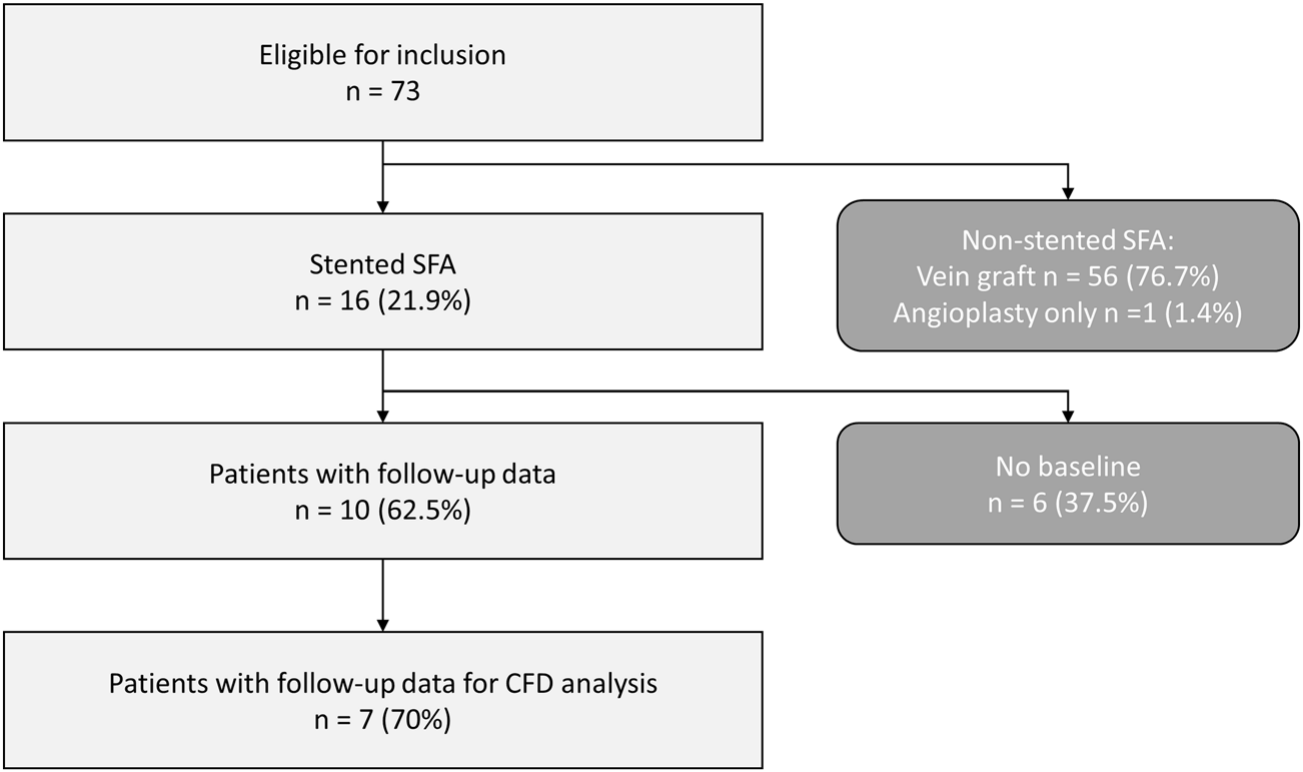
Patient flowchart presenting the complete study of the peripheral arteries.

Patients suffering from peripheral artery disease were screened at Malcom Randall VAMC (Gainesville, FL, USA) between 2007 and 2012 to identify those who were treated with self-expanding stents and consented to post-operative CT scan protocol (Fig. 2). Seven individuals (for a total of ten lesions, A-K, Table 1), treated with the EverFlex stent (EV3, Medtronic, Dublin, Ireland), presented the necessary CT and DUS data. Specifically, the CT and DUS data were gathered at 1-week (1W), 1-month (1M), 6-month (6M, except for one patient without usable CT data), and 1-year (1Y) post-operative follow-ups. The study was conducted in accordance with the principles of the Declaration of Helsinki and met the requirement of medical ethics. The study protocol was approved by the institutional review board at University of Florida and each participant provided the written consent.

**Table 1:** Clinical data.

Patient	Lesion	Age [years]	Stent length [mm]	Stented region length [mm]	Stent overlapping (length [mm])	Failure at 2 years	Coronary artery disease	Diabetes
**1**	**A**	56	60	60	No	No	No	No
**2**	**B**	60	120	120	No	No	yes	No
**3**	**C**	74	150	260	yes	Yes	No	No
			120		(10)			
**4**	**D**	61	100	200	Yes	Yes	Yes	No
			120		(20)			
**5**	**E**	73	120	305	Yes	Yes	Yes	Yes
			120		(20)			
			120		(35)			
**6**	**F**	64	30	30	No	No	Yes	Yes
	**G**	64	150	150	No	No	Yes	Yes
**7**	**H**	57	40	40	No	No	No	No
	**J**	57	150	255	Yes	Yes	No	No
			150		(45)			
	**K**	57	120	230	Yes	Yes	No	No
			150		(40)			

The baseline demographics and medical history (diabetes and coronary artery disease) of the selected patients are provided in Table 1, along with the intervention outcome (failure/success at post-operative 2-year), the stent length and the presence of stent overlapping (observed by visual inspection of CT images). The patients were all male, with an average age of 63.6±7.3 years (mean ± standard deviation).

### 3D Reconstruction and Hemodynamic Analysis

**Figure 3:**
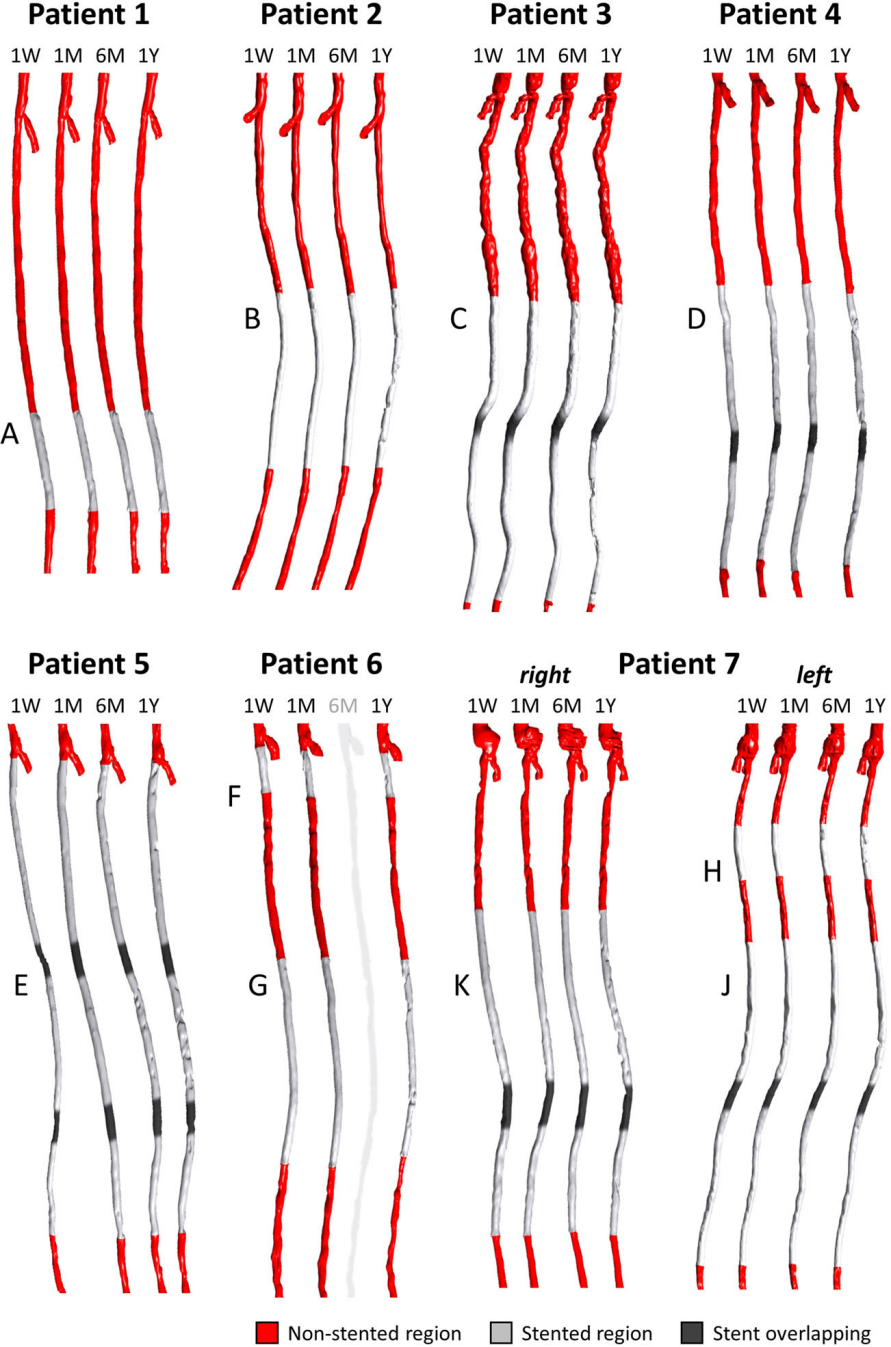
Three-dimensional stented superficial femoral artery models reconstructed from computed tomography images. The vessel geometries reconstructed at each follow-up are shown for each case under investigation except patient 6, which was characterized by unusable imaging data at 6-month follow-up. The common femoral artery bifurcation is included in all vessel models. For each patient presenting with overlapping stents, the overlapping region is indicated with a dark gray. The lesion name is indicated next to each stented region. 1W: 1-week; 1M: 1-month; 6M: 6-month; 1Y: 1-year.

Patient-specific SFA geometrical models (Fig. 3) were reconstructed from CT using a previously developed reconstruction method.^10^ In detail, a first raw 3D vessel reconstruction, including the common femoral artery bifurcation, was obtained by applying an active contour method, based on a level set algorithm.^10^ Then, the obtained geometrical model was automatically corrected in both the stented and non-stented regions through calibrated thresholds and freed from metallic artifacts and calcifications.^10^

The SFA models were used for both the morphological analysis (all the follow-ups) and the computation of hemodynamics (1W, 1M and 6M follow-ups). Regarding the CFD simulations, the models were discretized into tetrahedral elements considering curvature-based refinement and a prismatic boundary layer using ICEM CFD (v.18.2, Ansys Inc., Canonsburg, PA, USA). The element size was based on a previously performed mesh-independence study,^10^ resulting in a mesh cardinality ranging from 2,013,029 to 8,535,680 elements in the models A at 6M and K at 1W follow-up, respectively. Transient CFD simulations (*n* = 23) were performed using the commercial software Fluent (v.18.2, Ansys Inc.), based on the finite volume method. A patient-specific pulsatile flow waveform, derived from the patient’s DUS images, was applied to the inlet of the common femoral artery as a parabolic velocity profile. The sequence of peak velocities from the Doppler spectrum at the level of the common femoral artery was elaborated by applying a previously proposed algorithm^29^ that enabled the estimation of the patient-specific flow-rate. A flow-split of 0.67:0.33 was applied to the SFA and the profunda femoral artery, respectively.^20^ The no-slip condition was prescribed at the vessel walls, assumed as rigid. The blood was considered as an incompressible, homogeneous, non-Newtonian fluid with density of 1060 kg/m^3^ and viscosity described by the Carreau model. Regarding the solver settings, a pressure-based solver with a full implicit coupled scheme for the velocity-pressure coupling was adopted. Second order accuracy was chosen for pressure and momentum spatial discretization, and for time integration. Following a sensitivity analysis,^10^ the convergence for the continuity and momentum residuals was set to 5·10^−5^, and the cardiac cycle was discretized into 100 time steps (with time-step size in the range of [0.0061 - 0.0110] seconds, according to the patient-specific case). Further details about the CFD simulations are reported elsewhere.^10^

### Morphological and Hemodynamic Quantities of Interest

Lumen remodeling was quantified by calculating the lumen area change in the stented region (i.e. region of interest) as follows:

1 $$\Delta A_{i:i + 1} = \, A_{i} - A_{i + 1}$$

where *A* is the lumen area, and *i* is the specific follow-up. Accordingly, a positive lumen area change corresponds to inward remodeling (i.e. lumen reduction), whereas a negative lumen area change to outward remodeling (i.e. lumen enlargement). The fractional rate of lumen area change, normalized by the number of weeks occurred between two consecutive follow-ups, was computed for each time interval (i.e. first, 1W-1M; intermediate, 1M-6M; last, 6M-1Y).

Hemodynamics was investigated by analyzing the WSS vector field along the stented region. In addition to three well-known WSS-based descriptors, namely the time-averaged WSS (TAWSS), oscillatory shear index (OSI), and relative residence time (RRT), the following descriptors of WSS multidirectionality were quantified (Table 2, Fig. Suppl-1): transverse WSS (transWSS),^23^ i.e. the average of the WSS component perpendicular to the direction of the time-averaged WSS; cross flow index (CFI),^23^ i.e. the normalized transWSS; time-averaged axial WSS (TAWSSax),^24^ i.e. the average of the WSS component aligned with the tangent to the vessel centerline; time-averaged secondary WSS (TAWSSsc),^24^ i.e. the average of the WSS component along the secondary direction; and WSSratio,^24^ i.e. the ratio between the cycle-averaged magnitude of the secondary and the axial WSS components.

**Table 2:** Wall shear stress (WSS)-based hemodynamic descriptors.

Time-Averaged WSS	${\text{TAWSS}} = \frac{1}{T}\mathop {\smallint_{0}^{T}} \left| {{\mathbf{WSS}}} \right|dt$
Oscillatory Shear Index	${{\text{OSI}} = 0.5\left[ {1 - \left( {\frac{{\left| {\mathop {\smallint_{0}^{T}} \mathbf{WSS} dt} \right|}}{{\mathop {\smallint_{0}^{T}} \left| {\mathbf{WSS}} \right|dt}}} \right)} \right]}$
Relative Residence Time	${\text{RRT}} = \frac{1}{{{\text{TAWSS}} \cdot \left( {1 - 2 \cdot {\text{OSI}}} \right)}} = \frac{1}{{\frac{1}{T}\left| {\mathop {\smallint_{0}^{T}} \mathbf{WSS}dt} \right|}}$
Time-Averaged WSS_ax_ Magnitude	${\text{TAWSSax}} = \frac{1}{T}\mathop {\smallint_{0}^{T}} \left| {{\mathbf{WSS}}_{ax} } \right|dt$
Time-Averaged WSS_sc_ Magnitude	${\text{TAWSSsc}} = \frac{1}{T}\mathop {\smallint_{0}^{T}} \left| {{\mathbf{WSS}}_{sc} } \right|dt$
Transverse WSS	${\text{transWSS}} = \frac{1}{T}\mathop {\smallint_{0}^{T}} \left| {\mathbf{WSS} \cdot \left( {\varvec{n} \times \frac{{\mathop {\smallint_{0}^{T}} \mathbf{WSS}dt}}{{\left| {\mathop {\smallint_{0}^{T}} \mathbf{WSS}dt} \right|}}} \right)} \right|dt$
Cross Flow Index	${\text{CFI}} = \frac{1}{T}\mathop {\smallint_{0}^{T}} \left| {\frac{{\mathbf{WSS}}}{{\left| {\mathbf{WSS}} \right|}} \cdot \left( {\varvec{n} \times \frac{{\mathop {\smallint_{0}^{T}} \mathbf{WSS}dt}}{{\left| {\mathop {\smallint_{0}^{T}} \mathbf{WSS}dt} \right|}}} \right)} \right|dt$
WSS ratio	${\text{WSS}}_{ratio} = \frac{1}{T}\mathop {\smallint_{0}^{T}} \frac{{\left| {{\mathbf{WSS}}_{sc} } \right|}}{{\left| {{\mathbf{WSS}}_{ax} } \right|}}dt$

### Post-processing of Morphological and Hemodynamic Results

The morphological and CFD results were processed in order to (i) obtain a spatial match between the morphological and hemodynamic data and (ii) consider them as spatially independent, as described elsewhere.^8^ Briefly, the lumen area was extracted at 0.2 mm axially-spaced cross-sections and averaged every 1 mm in the axial direction. In this way, one-dimensional (1D) maps of the morphological descriptor were obtained. Similarly, the distributions of WSS-based descriptors were re-organized from three- to two-dimensional (2D) maps, with cells of 1 mm in the axial direction and 1 degree in the circumferential one (Fig. Suppl-2), by using the Vascular Modelling Toolkit (VMTK) (Orobix, Bergamo, Italy, http://www.vmtk.org/). To match the 1D morphological data, the WSS-based 2D maps were circumferentially-averaged, obtaining 1D maps (Fig. Suppl-2). The axial discretization of 1 mm was conservative with respect to the CT axial resolution, in the range of [0.87-0.98] mm.

The 1D maps were subjected to the spatial decorrelation analysis, and made mutually independent through the spatial decorrelation length (L_decorr_).^8^ L_decorr_ indicates the minimal length necessary to consider two data points as independent. Differently from our previous work,^8^ in which the hemodynamics was solely analyzed at baseline, the decorrelation length was computed here for each follow-up and lesion. Thus, considering separately each lesion, the most conservative L_decorr_ (range [2–10] mm) among 1W, 1M and 6M follow-ups was considered. Based on the identified L_decorr_, smaller but independent 1D maps of the morphological and hemodynamic data were obtained for each lesion and used in the subsequent statistical analyses.^8^

### Levels of Analysis

The analysis of results was conducted at two levels,^8^ namely (i) global level (i.e. between-stent level of analysis), by considering one representative averaged morphological and hemodynamic value for each lesion and each follow-up, and (ii) local level (i.e. within-stent level of analysis) by collecting, for each follow-up, the independent results contained in the lesion-specific arrays into a unique, combined array.

#### Morphological Progression

The lumen morphology of the entire stented regions was analyzed to investigate the lumen remodeling trajectory (whole-lesion analysis). Furthermore, the following additional investigations were considered:

i. Remodeling trajectory in fringe/mid portions of the lesion (segmental analysis). The stented region was subdivided into three groups: proximal and distal fringes (first and last two decorrelated points of the patient- and time-specific arrays), and the in-between (mid) portion.
ii. Remodeling trajectory in lesions treated with short and long stents. The lesions were subdivided into the following two groups: the lesions treated with short stents (range [40–120] mm, lesions A, B, F and H—Fig. 3, Table 1) and those treated with long stents (≥ 150 mm, considering together the multiple stents).
iii. Remodeling trajectory in overlapped/non-overlapped portions of the stents. Five lesions (lesions C-E, J and K—Fig. 3, Table 1) presenting with stent overlapping were analyzed by identifying three groups: the regions upstream or downstream from the overlapping and the region with stent overlapping.

#### Hemodynamics and ISR Progression

The relationship between hemodynamics and lumen remodeling over time was investigated in the entire stented regions to understand if:

i. the lumen area change *∆A*_*i: i+1*_ is associated to the WSS-based descriptors computed at the beginning of the time interval (*i*);
ii. the WSS-based descriptors at current follow-up (*i*) affects the lumen area at the subsequent follow-up *A*_*i+1*_;
iii. the change of WSS-based descriptors in the current time interval (*i*:*i+1*) influences the lumen area change in the subsequent time interval *∆A*_*i+1: i+2*_.

### Statistical Analysis

Data were presented as either mean ± standard deviation or median (interquartile range), depending on the distribution. The normality of the distributions was evaluated using Kolmogorov-Smirnov test.

To examine the lumen remodeling, the comparison of the lumen area distributions at each follow-up and of the fractional rate of lumen area change in the different time intervals was performed. The different groups were compared using analysis of variance or Kruskal-Wallis tests. Then, the individual distributions were tested through Mann-Whitney U or Friedman tests. The linear regression between the lumen area change in each time interval and the lumen area at the beginning of that time interval was also applied.

To measure the direction and strength of the association between WSS-based descriptors and lumen remodeling, the Spearman’s rank-order correlation coefficient was considered. Moreover, to evaluate the probability that luminal surface areas exposed to disturbed shear can successfully identify corresponding regions with large lumen area change, the positive predictive value (PPV) was computed. To do so, objective thresholds of disturbed shear stress were defined by combining the follow-up and lesion-specific WSS-based data.^8^ The 33^th^ percentile was identified for TAWSS, TAWSSax and TAWSSsc; the 66^th^ percentile was chosen for the other WSS-based descriptors. The percentage of luminal surface area exposed to disturbed shear stress (lower or higher than the given thresholds, depending on the WSS-based descriptor) was computed for each follow-up. The lumen area change was distinguished in low/high according to the 66^th^ distribution percentile.

To integrate the previous investigations, linear mixed models were also built to evaluate the relationship between the lumen area change and one of the WSS-based descriptors, plus clinical data (i.e. patient’s age, presence of comorbidities like diabetes and coronary artery disease, and failure at 2-year post-intervention) and the categorical variable of follow-up time. The analyses were performed by considering the patient as the random factor to account for multiple measures.

The statistical analyses were performed using GraphPrism (v.8.3.1, GraphPad Software) and SAS (v.9.4, SAS Institute). A two-tailed, *p*-value < 0.05 was considered to be statistically significant.

## Results

### Morphological Progression: Whole-Lesion Analysis

**Figure 4:**
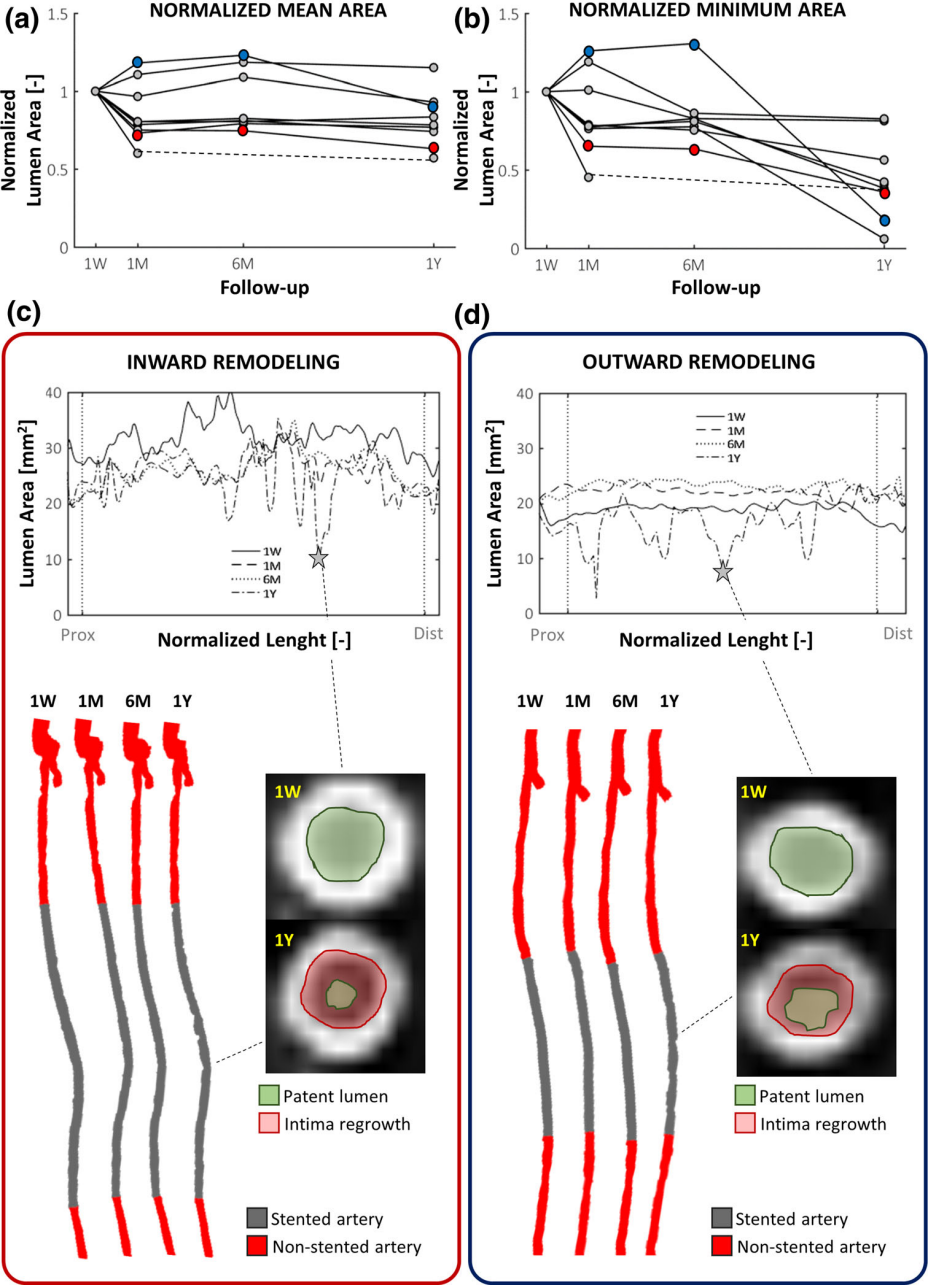
Lumen remodeling trajectory of all investigated cases. (**a-b**) Trends over time of the mean and minimum lumen area, normalized by the initial area at 1W (global level of analysis). Two representative cases (lesions K and B in Table 1) presenting with inward (red dots) remodeling or outward (blue dots) remodeling in the first time-interval (1W-1M) are chosen as example. (**c-d**) Details of the two explanatory cases subjected to inward or outward remodeling: at the top, the lumen area at each follow-up along the normalized lesion length, from the proximal (left) to distal edge (right); at the bottom, the corresponding reconstructed anatomies at different follow-ups, from 1W (left) to 1Y (right) models; alongside, explanatory CT images of vessel cross-sections (at 1W and 1Y), which were subjected to focal lumen re-narrowing at 1Y. The CT scans were colored to indicate the initial and final patent lumen (in green) and the abnormal intimal regrowth found at 1Y (in red). **1W**: 1-week; **1M**: 1-month; **6M**: 6-month; **1Y**: 1-year; **prox**: proximal region of the stented portion; **dist**: distal region.

The global trends of mean lumen area over time, normalized by the 1W value, are shown in Fig. 4a for all lesions. The main changes of mean lumen area occurred in the first time interval in the majority of cases. All lesions but two exhibited a decrease of mean lumen area (i.e. inward remodeling) in the first time interval followed by a more stable trend along the subsequent time intervals. When considering the normalized minimum lumen area (Fig. 4b), a wider variety of trends was observable. Four lesions were characterized by a severe reduction of the minimum lumen area in the last time interval. All lesions, including those presenting with outward remodeling in the first time interval, ended up in a smaller minimum lumen area at 1Y as compared to 1W. As highlighted by the explanatory examples of Fig. 4c, d (lesions K and B, presenting with inward and outward remodeling in the first two time intervals, respectively), a focal re-narrowing took place in the last time interval, thus explaining the observed small minimum lumen area at 1Y. The focal re-narrowing was caused by the abnormal intima regrowth, as visually detected in the 1Y CT images.

**Figure 5:**
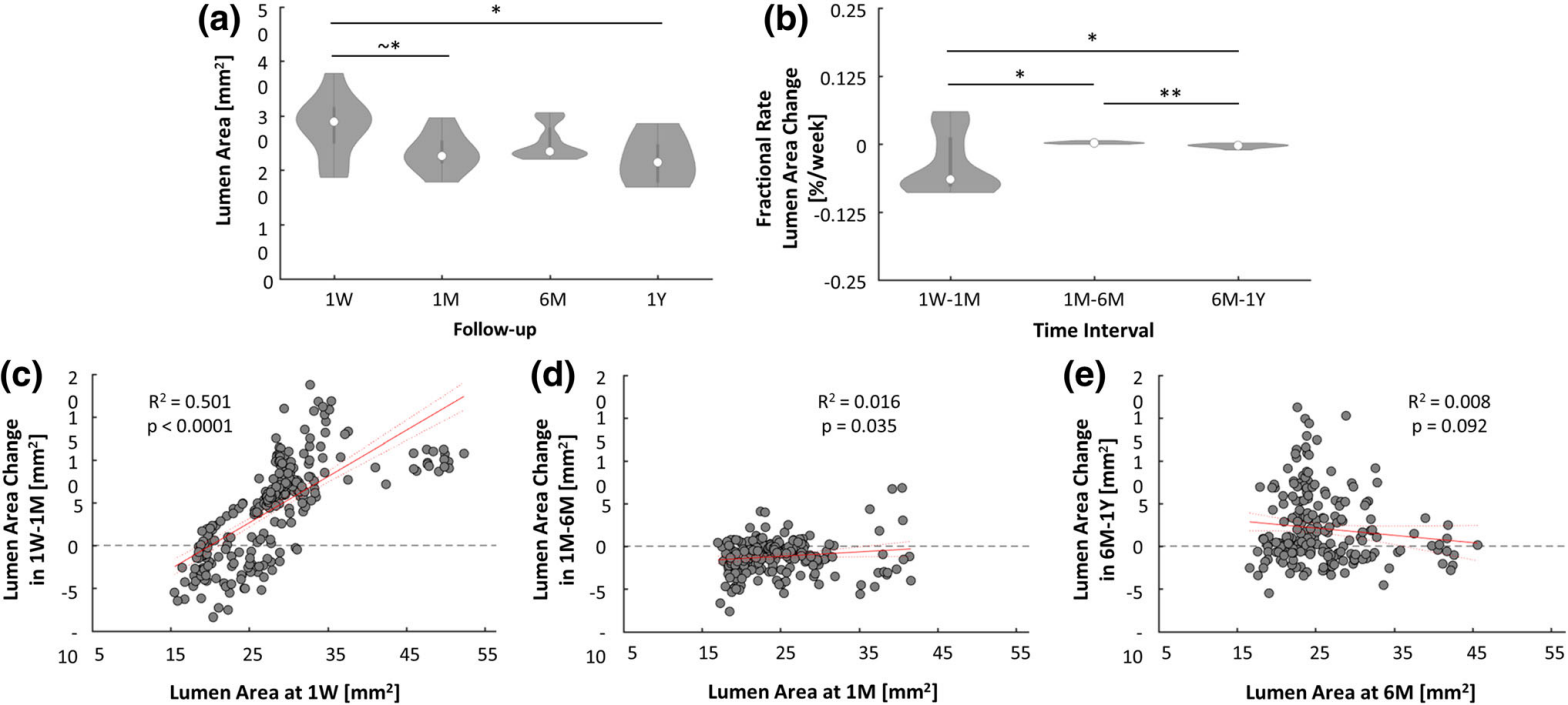
(**a**) Distributions of the lumen area of the lesion at different follow-ups (global level of analysis). (**b**) Fractional rate of lumen area change of the lesion computed in the different time intervals (global level of analysis). (**c-e**) Results of the linear regression between the lumen area change at each specific time interval and the lumen area at the beginning of that time interval (local level of analysis). The confidence bounds are shown in dashed red line. **1W**: 1-week; **1M**: 1-month; **6M**: 6-month; **1Y**: 1-year; ^~^*p = 0.05; *p < 0.05; **p < 0.01.

The comparison of the mean lumen area distributions at the different follow-ups at the global level (Fig. 5a) revealed a nearly statistically significant difference between the distributions at 1W and 1M (28.91 [6.61] vs. 22.60 [4.11] mm^2^, *p* = 0.0539) and a significant difference between the distributions at 1W and 1Y (28.91 [6.61] vs. 21.43 [6.91] mm^2^, *p* = 0.0140). This suggests that the lumen area reduced mainly in the first time interval and that the final lumen area was significantly different from the 1W condition. The findings were confirmed also at the local level, with the difference that, due to the large dimension of the dataset, statistically significant differences emerged in all time intervals (Fig. Suppl-3a).

Likewise, at the global level, the fractional rate of lumen area change occurring in the first time interval was significantly different from that in the intermediate (*p* = 0.0343) and last (*p* = 0.0434) time intervals (Fig. 5b). A strong statistically significant difference in the fractional rate of change between the intermediate and late time interval was also found (*p* = 0.0011), though the absolute difference was negligible. As in the previous comparison of the lumen area distributions, the local-level results confirmed the global-level results (Fig. Suppl-3B).

The linear regression between the lumen area change ∆A_i:i+1_ and the area A_i_ is reported in Fig. 5c–e. A strong positive correlation was found only in the first time interval, indicating that the larger the lumen area at 1W, the larger the lumen inward remodeling in that time interval. No strong association emerged for the other time intervals.

### Morphological Progression: Additional Analyses

In general, the local-level analysis confirmed the findings at the global level with the advantage of enlarging the number of data points in the case of short vessel portions. Hence, only the local results are reported here. A description of the global results is presented in the supplementary materials (Figs. Suppl-4-6).

**Figure 6:**
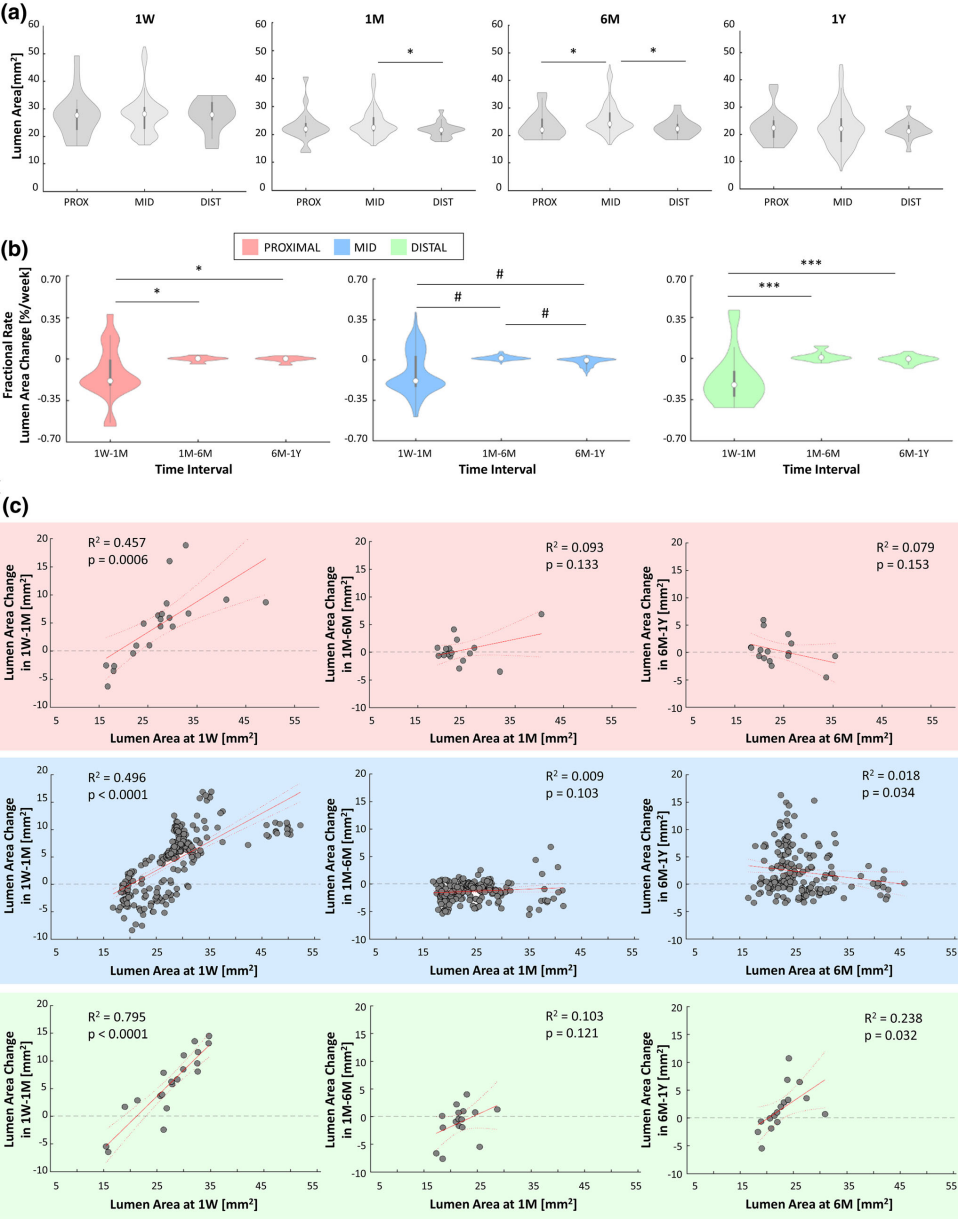
Results of the morphological remodeling considering the proximal, mid and distal segments. (**a**) Comparisons at the local level of the lumen area distributions in the segments at each follow-up. (**b**) Local results of the fractional rate of lumen area change in the three time intervals (1W-1M, 1M-6M and 6M-1Y) for the proximal, mid and distal segments. (**c**) Local analysis of the linear regression for each segment between the lumen area change in the time interval and the lumen area at the beginning of that period. **1W**: 1-week; **1M**: 1-month; **6M**: 6-month; **1Y**: 1-year; **PROX**: proximal; **DIST**: distal; *p < 0.05; ***p < 0.001; ^**#**^*p* < 0.0001.

**Figure 7:**
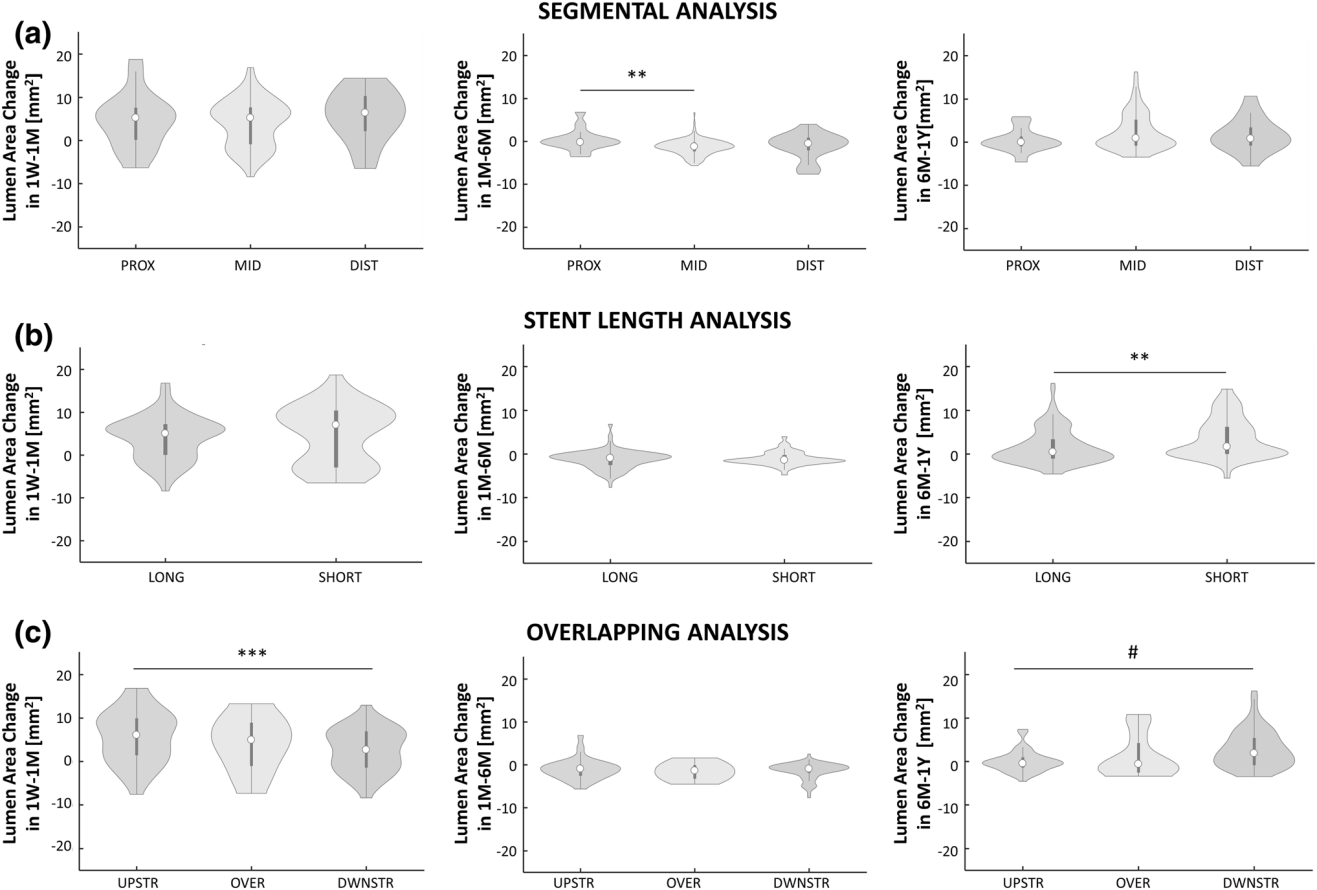
Local analysis of the lumen area change distributions in the three time intervals 1W-1M, 1M-6M and 6M-1Y in case of (**a**) segmental analysis: comparison of the lumen area change distributions between the proximal, mid and distal segments; (**b**) analysis of the impact of stent length: comparison of the lumen area change distributions between the long and short devices; and (**c**) analysis of the impact of stent overlapping: comparison of lumen area change distributions among the segment upstream from the overlapping, the portion with overlapped, and the segment downstream from the overlapping. **1W**: 1-week; **1M**: 1-month; **6M**: 6-month; **1Y**: 1-year; **PROX**: proximal; **DIST**: distal; **UPSTR**: upstream; **DWSTR**: downstream; **OVER**: overlapping; **p < 0.01; ***p < 0.001, ^**#**^*p* < 0.0001.

The lumen remodeling was slightly segment specific. In particular, significant differences emerged between the mid and distal segments at 1M (22.38 [4.93] vs. 21.53 [2.60] mm^2^, *p* = 0.0229), the proximal and mid segments at 6M (21.93 [5.42]vs. 24.13 [5.60] mm^2^, *p* = 0.0362), and mid and distal segments at 6M (24.13 [5.60] vs. 22.33 [3.37] mm^2^, *p* = 0.0122) (Fig. 6a). The fractional rate of lumen area change in the different segments (Fig. 6b) was similar to that of the entire lesion (Fig. 5b), indicating that the fastest and highest lumen remodeling occurred in the first time interval throughout the lesion. Furthermore, it is noteworthy that in all the segments the lumen area A_1W_ was positively correlated with the lumen area change ∆A_1W:1M_ (Fig. 6c). The lumen area change in the proximal segment was significantly smaller than that in the mid segments in the intermediate time interval (-0.21 [1.50] vs. -1.24 [1.98] mm^2^, *p* = 0.0030) (Fig. 7a).

No significant differences in lumen area changes were found between short and long stents except in the last time interval, in which the short stents presented a slightly higher inward remodeling than the long stents (1.67 [6.09] vs. 0.44 [4.32] mm^2^, *p* = 0.0017, Fig. 7b).

A significant difference between the segments upstream and downstream of the overlapping portion emerged in the first and last time intervals (*p* = 0.0003 and *p* < 0.0001, respectively) (Fig. 7c). In the first time interval, the inward remodeling was larger in the upstream segment compared to the downstream one (6.07 [8.40] vs. 2.66 [8.23] mm^2^, *p* = 0.0003). In the last time interval, the downstream segment had a larger lumen reduction than the other portions, significantly different compared to the upstream segment (1.87 [6.14] vs. -0.47 [2.23] mm^2^, *p* < 0.0001).

### Hemodynamics and ISR Progression

**Figure 8:**
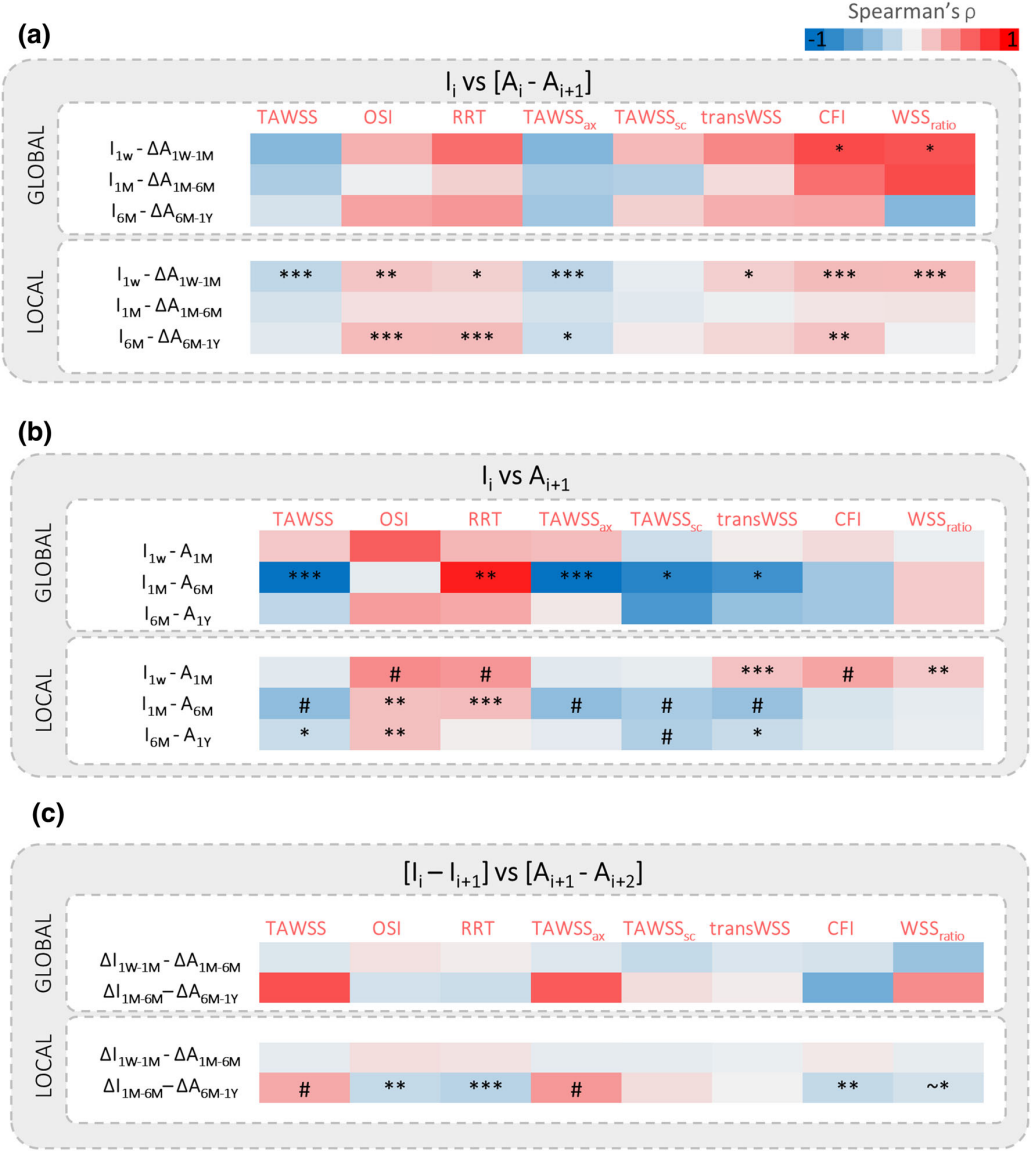
Spearman’s rank correlations at the global and local level of analysis between the morphological and hemodynamic data. (**a**) Quantification of the association between the lumen area change in the current time interval and the WSS-based descriptors computed at the beginning of that time interval. (**b**) Quantification of the association between the WSS-based descriptors at current follow-up and the lumen area at consecutive follow-up. (**c**) Quantification of the association between the change of WSS-based descriptors in the current time interval and the lumen area change in the following time interval. **I**: WSS-based descriptor; **A**: lumen area; ***i***: current time instant; **1W**: 1-week; **1M**: 1-month; **6M**: 6-month; **1Y**: 1-year. ^~^*p = 0.05; ^*^p < 0.05; ^**^p  < 0.01; ^***^p < 0.001; ^**#**^*p* < 0.0001.

The results of the Spearman’s correlation between the WSS-based descriptors and the morphological progression are summarized in Fig. 8. Considering the possible link between the hemodynamics at the beginning of the time interval and the lumen area change in that time interval (Fig. 8a), at the global level, a strong positive correlation was found between CFI_1W_ and lumen area change ∆A_1W:1M_ (i.e. for high CFI, an inward remodeling occurred; ρ = 0.697, *p* = 0.0311), and between WSSratio_1W_ and lumen area change ∆A_1W:1M_ (ρ = 0.661, *p* = 0.0440). A negative trend was found between TAWSS_1W_ and lumen area change ∆A_1W:1M_ (i.e. for low TAWSS, an inward remodeling occurred), and a positive trend between OSI_1W_ and ∆A_1W:1M_, and between RRT_1W_ and ∆A_1W:1M_. These findings were statistically supported by the local-level analysis. All the WSS-based descriptors at 1W but the TAWSSsc_1W_ were correlated with the lumen area change ∆A_1W:1M_. Furthermore, the OSI_6M_, RRT_6M_ and CFI_6M_ were positively associated with ∆A_6M:1Y_ (ρ = 0.223 with *p* = 0.0004, ρ = 0.228 with *p = *0.0006, ρ = 0.202 with *p = *0.0024, respectively), while the TAWSSax_6M_ was negatively associated with ∆A_6M:1Y_ (ρ = -0.157, *p * = 0.0185).

Considering the effect of each WSS-based descriptor on the lumen area at the following follow-up (Fig. 8b), at the global level, statistically significant associations were found only for the intermediate time interval. These results were affected by the small lumen area change in that time interval, in which a similar lumen morphology at 1M and 6M was present, and thus reflect the known relationship between lumen geometry and WSS-based descriptors (e.g. with higher TAWSS in the case of smaller lumen diameter). These results were confirmed at the local level. Furthermore, the OSI was the only WSS-based descriptor presenting a positive correlation with the lumen area at all the subsequent follow-ups (ρ = 0.423 with *p* < 0.0001 at 1W, ρ = 0.212 with *p* = 0.0014 at 1M, and ρ = 0.212 with *p* = 0.0015 at 6M).

Considering the possible link between the change of the WSS-based descriptor during a specific time interval and the lumen area change in the subsequent time interval (Fig. 8c), at the global level, a nearly significant strong positive correlation between ΔTAWSS_1M:6M_ and lumen area change ∆A_6M:1Y_ was found (ρ = 0.670, *p* = 0.0689). This indicates that a reduction of TAWSS in the time interval 1M-6M could be associated to inward remodeling in the following time interval (6M-1Y). No other statistically significant associations were detected. However, the global trends were confirmed by the local analysis, which showed significant correlations for ΔTAWSS_1M:6M_ (ρ = 0.306, *p* < 0.0001), ΔOSI_1M:6M_ (ρ = -0.183,* p* = 0.0061), ΔRRT_1M:6M_ (ρ = -0.232, *p* = 0.0005), ΔTAWSSax_1M:6M_ (ρ = 0.372, *p* < 0.0001), and ΔCFI_1M:6M_ (ρ = -0.190, *p* = 0.0045) with the lumen area change ∆A_6M:1Y_.

**Figure 9:**
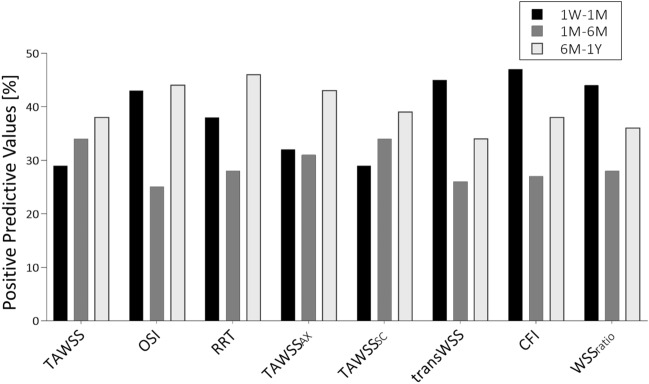
Results in terms of positive predictive value (PPV) for the local analysis, indicating the capability of the abnormal WSS-based descriptors to identify regions with extensive lumen area reduction in the three time intervals of interest: 1W-1M, 1M-6M and 6M-1Y. **1W**: 1-week; **1M**: 1-month; **6M**: 6-month; **1Y**: 1-year.

The predictive capacity of the WSS-based descriptors was evaluated by quantifying the PPV for each time interval (Fig. 9). In the first time interval, the PPV of CFI, which was the WSS-based descriptor characterized at the global level by the highest correlation coefficient with the lumen area change, was the highest (47.3%). Moreover, the PPV of OSI, transWSS and WSSratio was higher than 40% (43.5%, 44.9% and 44.1%, respectively). In the intermediate time interval, characterized by small lumen morphological changes, the PPV of each WSS-based descriptor was lower (<40%). In last time interval, only the PPV of OSI, RRT and TAWSSax was higher than 40% (44.0%, 46.2% and 43.4%, respectively).

Regarding the linear mixed models, significant result was obtained for the CFI (F = 6.22, *p* = 0.0248), consistently with the global results of correlation between WSS-based descriptors and morphological progression.

## Discussion

In this study, the progression of ISR in stented SFAs was investigated over 1Y follow-up period. Patient-specific CFD simulations were performed at multiple follow-ups to elucidate the possible link between altered hemodynamics and ISR progression. As main findings, in human stented SFAs (i) the first month post-intervention was the most dynamic in terms of lumen remodeling, presenting the largest lumen area changes; (ii) focal re-narrowing frequently occurred in the last time interval of investigation (6M-1Y); (iii) the lumen remodeling was similar along the lesion length, with slight region-specific differences; (iv) multidirectional WSS seemed to be involved in the process of lumen remodeling throughout the first year post-intervention, while low WSS impacted the early phases.

### Morphological Progression

The morphological analysis revealed that the fastest lumen reduction occurred in the first post-operative time interval in most investigated lesions. This result is consistent with the current knowledge on ISR, according to which the inward remodeling, following a phase of inflammation, becomes relevant starting from the third week after endovascular intervention.^4^,^12^ In the intermediate time interval, the lumen area change was markedly smaller, in accordance with the concept that the healing of the arterial wall and the stabilization of the neointima can be reached at 1M.^17^ However, in the last time interval, the lumen area change was not negligible, even though smaller as compared to the first time interval. Short portions of the stented regions presented focal lumen re-narrowing, resulting in a severe reduction of the minimum lumen area in four lesions out of ten. This finding is consistent with the observation that the largest lumen remodeling in SFAs occurs at 1Y post-intervention.^19^

Overall, similar results were found by analyzing the ISR progression of both the entire lesions and segments of the lesions. Specifically, the lumen remodeling trajectory (i.e. the global trends of mean lumen area over time), the lumen area distributions at the different follow-ups and the fractional rates of change in the proximal, mid and distal segments were comparable to those of the entire lesions. At the local level, significantly higher mean lumen area was found in the mid segment with respect to the distal one at 1M and both the proximal and distal segments at 6M. A direct comparison of these results with other studies is not straightforward. In animal studies on coronary stents, significant differences in lumen area were found between the fringes and mid segments at 1M,^31^ or along the stented region at 14 days post-intervention.^35^

The present work proves the importance of observing the entire remodeling trajectory of ISR in human arteries, by focusing on human stented SFAs. No longitudinal data on ISR in human stented SFAs are currently available to be directly compared with the findings of the present study. To date, most studies^1^,^31^ investigating ISR in femoral arteries (i) are based on animal models, (ii) consider the lumen remodeling for a short period post-intervention (usually 1M follow-up, because of the sacrifice of the animals for the follow-up measurement), and (iii) exclude the presence of a prior atherosclerotic lesion that may complicate the ISR process, as the devices were implanted in swine healthy arteries. Similar considerations are valid for animal studies related to ISR in coronary arteries, characterized by a short follow-up period (2 weeks or 1M).^6^,^25^,^35^

A similar approach to morphological analysis was applied in a previous longitudinal study quantifying the lumen remodeling trajectory over 1Y follow-up period in patients treated with infra-inguinal vein-graft.^15^ A direct comparison between the lumen remodeling trajectories is not possible because the biological processes leading to ISR in stented SFAs and restenosis in vein grafts are different. However, it is worth noting that the most dramatic lumen area change was also observed at 1M in vein grafts.

### Impact of stent length and overlapping

The length of a treated lesion is an important clinical aspect since it varies considerably and ranges from short (<150 mm) to considerably long lesions (>150 mm).^3^ Accordingly, the lumen remodeling trajectory was here explored in two groups of lesions treated with either short or long stents. No significant differences in lumen area changes emerged between the groups in the first time interval, in accordance with the finding that initial outcomes of the EverFlex stent were independent of the device length.^30^ However, in the longer period, significant differences emerged between the groups in the time interval 6M-1Y. In particular, the short stents were characterized by a slightly higher inward remodeling than the long devices.

Multiple overlapping stents are implanted to cover the long lesions. Contradictory results were reported for the coronary^16^ and carotid^28^ arterial beds, suggesting that the vascular location might have an impact on remodeling in the case of stent overlapping. For the SFA, a vascular region widely subjected to dynamic forces,^18^ the presence of multiple stents may generate areas of greater rigidity and, consequently, of more frequent ruptures and failure.^2^ As recently reported,^5^ the long single-stent strategy seems more appropriate than a multiple-stent strategy. In the present work, significant differences in lumen area changes were found between the segments upstream and downstream from the overlapping portion in the time intervals 1W-1M and 6M-1Y. Moreover, in both time intervals, the overlapping portion showed a smaller inward remodeling than the other two regions. These findings suggest that stent overlapping in SFAs may also contribute to a region-specific lumen remodeling.

### Hemodynamics and ISR Progression

In addition to the wall injury provoked by angioplasty and subsequent stent implantation, which has been recognized to trigger neointimal hyperplasia,^7^,^31^ the altered hemodynamics post-intervention seems to promote and modulate the inward lumen remodeling over time.^27^ In the context of lower-limb arteries, recent studies investigated the relationship between altered hemodynamics at baseline and the occurrence of restenosis at 6M after angioplasty or stenting in human femoropopliteal arteries,^13^ or at 1Y in human SFAs treated with self-expanding stents (same dataset of the present work).^8^ In particular, a negative correlation between TAWSS at baseline and lumen reduction at 1Y was found in stented SFAs.^8^ The present study extended the previous research by analyzing the impact of altered hemodynamics on the progression of lumen remodeling over 1Y follow-up period.

The hemodynamic results confirmed an inverse association between TAWSS and the lumen area reduction at each time interval under consideration. Furthermore, they highlighted the role of the multi-directional WSS in the lumen remodeling post-intervention. Indeed, in contrast to other vascular regions, such as coronary arteries,^11^ in the SFAs the WSS is highly multidirectional mainly because of their triphasic inflow curve (with a short phase with reverse flows^10^), high flow-rate (mean flow up to 400 mL/min at rest^10^) and tortuosity.^22^ Here, consistent trends between the multi-directional WSS-based descriptors and lumen area reduction were found in all the time intervals. Significant associations emerged at the local level for all multi-directional WSS-based descriptors but the TAWSSsc in the first time interval. Interestingly, these associations were present also at the global level for CFI and WSSratio.

Additionally, the predictive capacity of the WSS-based descriptors was evaluated. Promising PPVs (>40%) were found in the first and last time intervals. In particular, the CFI showed the highest predictive capacity (~47%) in the first time interval. Conversely, lower PPVs values were found in the intermediate time interval, as a result of the small lumen morphological changes.

### Limitations and Conclusion Remarks

This study is not without limitations. The dimension of the clinical dataset, both at the global level, with a low number of lesions, and at the local level, with high variability of lumen area along the vessel, affected the statistical power of the models. However, by combining the different levels of investigation, it was possible to better interpret the outcomes, being the global results supported by the consistent local ones. Similarly to previous works,^8^,^13^ the SFA models did not include the stent strut geometry, limited by the CT resolution.^10^ The outward remodeling detected in two lesions (B and E) could be explained by both the CT resolution, affecting the vessel reconstruction, and a prolonged stent expansion over time favored by calcifications (as visible from CT). The CFD simulations were performed under the assumptions of rigid walls and straight leg configuration. In the future, a fully personalization of the models may be achieved by considering the lower limb movement through the implementation of a recently proposed methodology.^9^

In conclusion, despite the limited number of available lesions, this study gained deeper insights into ISR progression in human stented SFAs over 1Y follow-up period, disclosing the importance of long-term follow-ups in the investigation of ISR, without which information on the procedural outcomes as well as on the hemodynamic promoting factors of ISR might be lost. The present findings revealed that the lumen remodeling of human stented SFAs is a dynamic process characterized by high lumen area change during the first month post-intervention and possible focal re-narrowing in the last analyzed time interval. Furthermore, the hemodynamic results suggested the multidirectional WSS is an important biomechanical factor involved in the ISR process, which modulates the inward lumen remodeling of the lesions over time.

## Supplementary Information

Below is the link to the electronic supplementary material.

Supplementary material 1 (PDF 1,207 kb).

## Abbreviations

CFD: Computational fluid dynamics
CT: Computed tomography
DUS: Doppler ultrasound
WSS: Wall shear stress
ISR: In-stent restenosis
SFA: Superficial femoral artery
1W: 1-week
1M: 1-month
6M: 6-month
1Y: 1-year
TAWSS: Time-averaged wall shear stress
OSI: Oscillatory shear index
RRT: Relative residence time
TAWSSax: Axial component of time-averaged wall shear stress
TAWSSsc: Secondary component of time-averaged wall shear stress
transWSS: Transverse wall shear stress
CFI: Cross flow index
WSSratio: Wall shear stress ratio
PPV: Positive predictive value
1D: One-dimensional
2D: Two-dimensional
3D: Three-dimensional
