# Supplementary material for: In-Stent Restenosis Progression in Human Superficial Femoral Arteries: Dynamics of Lumen Remodeling and Impact of Local Hemodynamics
Source: Ann Biomed Eng. 2021 Apr 29;49(9):2349–64. doi: 10.1007/s10439-021-02776-1 (PMC8455500; doi:10.1007/s10439-021-02776-1)
Supplement: Supplementary file 1 — Supplementary material 1 (PDF 1,207 kb). [file 10439_2021_2776_MOESM1_ESM.pdf]

In-stent restenosis progression in human superficial femoral arteries: dynamics of lumen remodeling and impact of local hemodynamics

SUPPLEMENTARY MATERIAL

Monika Colombo[1], Yong He[2], Anna Corti[1], Diego Gallo[3], Federica Ninno[1,4], Stefano Casarin[5,6,7], Jared M Rozowsky[2], Francesco Migliavacca[1], Scott Berceli[2,8], Claudio Chiastra[1,3*]

1. Laboratory of Biological Structure Mechanics (LaBS), Department of Chemistry, Materials and Chemical Engineering "Giulio Natta", Politecnico di Milano, Milan, Italy
2. Department of Surgery, University of Florida, Gainesville, FL, USA
3. PoliTo[BIO]Med Lab, Department of Mechanical and Aerospace Engineering, Politecnico di Torino, Turin, Italy
4. Department of Medical Physics and Biomedical Engineering, University College of London, London, United Kingdom
5. Department of Surgery, Houston Methodist Hospital, Houston, TX, USA
6. Center for Computational Surgery, Houston Methodist Research Institute, Houston, TX, USA
7. Houston Methodist Academic Institute, Houston, TX, USA
8. Malcom Randall VAMC, Gainesville, FL, USA

***Address for correspondence:**

Claudio Chiastra, PhD

PoliTo[BIO]Med Lab

Department of Mechanical and Aerospace Engineering

Politecnico di Torino

Corso Duca degli Abruzzi, 24

10129 Turin, Italy ORCID: 0000-0003-2070-6142

E-mail: claudio.chiastra@polito.it

SUPPLEMENTARY METHODS

Hemodynamic descriptors

Multiple cardiac cycle-averaged descriptors, which take into account the pulsatile nature of the femoral blood flow-rate, were explored in the present work. These WSS-based descriptors adopted to characterize disturbed flow involving either magnitude-based indicators (i.e. TAWSS, OSI, RRT), or direction-based indicators (i.e. transWSS, CFI, TAWSSax, TAWSSsc, and WSSratio)[S1-3] (Table 2). In detail, as depicted in Figure 1-Suppl, along the vessel centerline C(s), where s is the curvilinear abscissa, it is possible to define a local reference system composed by the tangent vector to the centerline (**C'**), the vector perpendicular to **C'** and directed toward a generic point on the luminal surface (**R**), and the vector orthogonal to **C'** and **R** (namely, **S**). Through this local reference system, the **WSS** vector can be projected along the direction of the vessel centerline represented by **C'** (**WSSax**, the axial component, green arrow in Figure Suppl-1) and along the direction perpendicular to the arterial centerline represented by **S** (**WSSsc**, the secondary component, blue arrow in Figure Suppl-1). Then, their cardiac cycle-averaged quantities were computed, obtaining TAWSSax and TAWSSsc. Additionally, the ratio of the magnitudes of the secondary to axial components of the **WSS** vector, namely WSSratio, was introduced to determine its predominant direction.

Post-processing of the hemodynamic results

To spatially match the one-dimensional (1D) morphological data, as illustrated in Figure Suppl-2, the three-dimensional (3D) distributions of the WSS-based descriptors were transformed into 1D maps by means of the following procedure: (i) re-organization of the 3D distributions into two-dimensional (2D) maps with cells of 1 mm in the axial direction and 1 degree in the circumferential one using the

Vascular Modelling Toolkit (VMTK) (Orobix, Bergamo, Italy, http://www.vmtk.org/); (ii) circumferential averaging of the WSS-based 2D maps to obtain the final 1D maps.

SUPPLEMENTARY RESULTS

Morphological progression: whole-lesion analysis

The comparison of the mean lumen area distributions at the different follow-ups at the local level (Figure Suppl-4a) revealed significant differences among all the distributions, due to the large dimension of the dataset. Specifically, the following differences were found: 1W-1M (27.89 [7.83] vs. 22.31 [4.92] mm^2, $p < 0.0001$), 1W-6M (27.89 [7.83] vs. 24.01 [5.36] mm^2, $p < 0.0001$), 1W-1Y (27.89 [7.83] vs. 22.00 [7.51] mm^2, $p < 0.0001$), 1M-6M (22.31 [4.92] vs. 24.01 [5.36] mm^2, $p < 0.0001$), 1M-1Y (22.31 [4.92] vs. 22.00 [7.51] mm^2, $p < 0.0001$), and 6M-1Y (24.01 [5.36] vs. 22.00 [7.51] mm^2, $p = 0.0027$).

In Figure Suppl-4b, the results for the fractional rate of change are reported. As before, all the time intervals are characterized by significant differences (1W-1M vs. 1M-6M with $p < 0.0001$, 1W-1M vs. 6M-1Y with $p < 0.0001$, and 1M-6M vs. 6M-1Y with $p < 0.0001$).

Morphological progression: additional analyses

Regarding the segmental analysis at the global level, the lumen remodeling in different vessel segments (i.e. proximal, mid and distal segments of the SFA lesions) was studied by comparing the lumen area distributions at each follow-up (Figure Suppl-5a), in which no significant differences were found, and the fractional rate of lumen area change in the three time intervals under investigation (Figure Suppl-5b), in which only the mid segment of the stented region presented a significant

difference of the lumen area change between the time intervals 1M-6M and 6M-1Y (p = 0.0006). The trends over time of the mean and minimum lumen area, normalized by the initial area at 1W, of each segment (Figure Suppl-6a) were characterized by the largest remodeling in the time interval 1W-1M. Afterwards, both the mean and minimum lumen area reach a plateau. Regarding the global temporal trend of the normalized minimum area, the mid segment was characterized by the presence of the lowest values at 1Y. Globally, the remodeling in the mid portion was higher in the first and last time intervals. The lumen area change distributions in the three time intervals 1W-1M, 1M-6M and 6M-1Y in case of the different segments were compared (Figure Suppl-6a). In particular, the lumen area change of the proximal segment significantly differed in the time interval 1M-6M (0.45 [1.99] vs. -0.57 [2.18] mm^2, p = 0.0148), and nearly significantly differed in the time interval 6M-1Y (-0.36 [2.65] vs. 0.99 [4.28] mm^2, p = 0.0650) from that of the distal one.

Regarding the analysis of the impact of stent length (Figure Suppl-6b) and stent overlapping (Figure Suppl-5c) at the global level, no significant differences in terms of lumen area change distribution were found.

SUPPLEMENTARY FIGURES

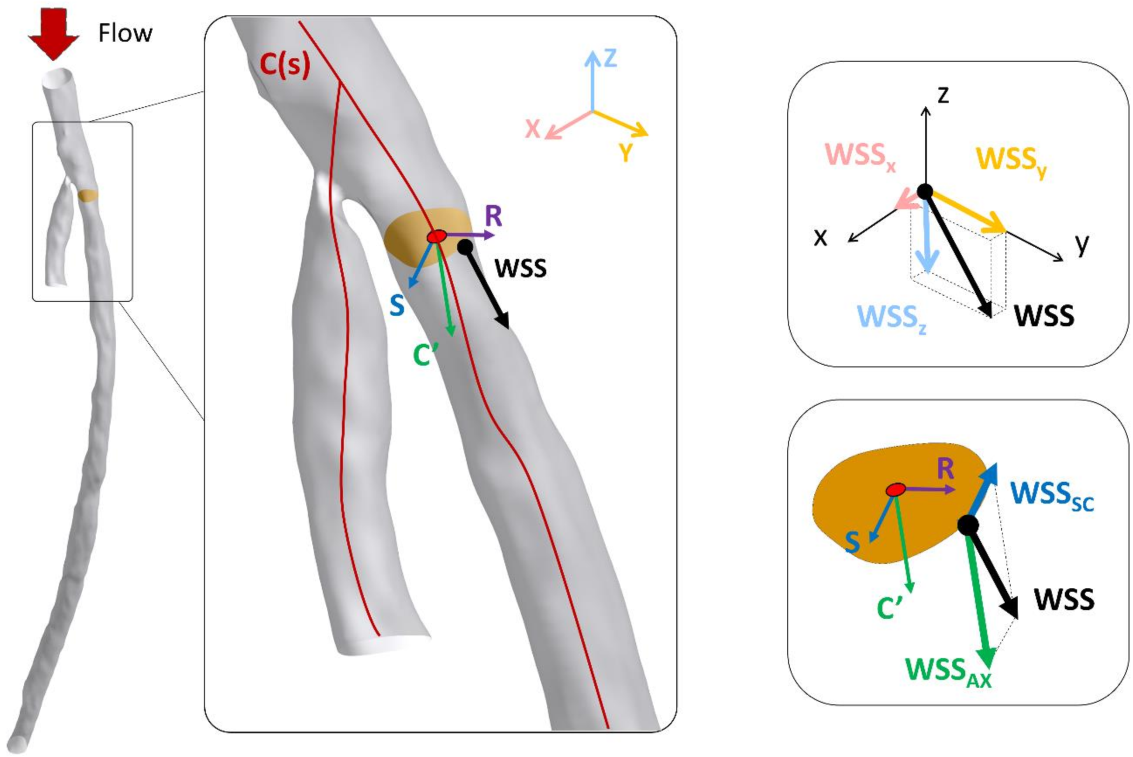

Figure Suppl-1 – Representative femoral artery model with its centerline (red line) and instantaneous **WSS** vector (black arrow) on a specific point of the luminal surface. The Cartesian **WSS** vector components (yellow, pink and light blue arrows), as well as the projection along the centerline (axial, green arrow) and its perpendicular directions (secondary, blue arrow) are presented in the boxes on the right.

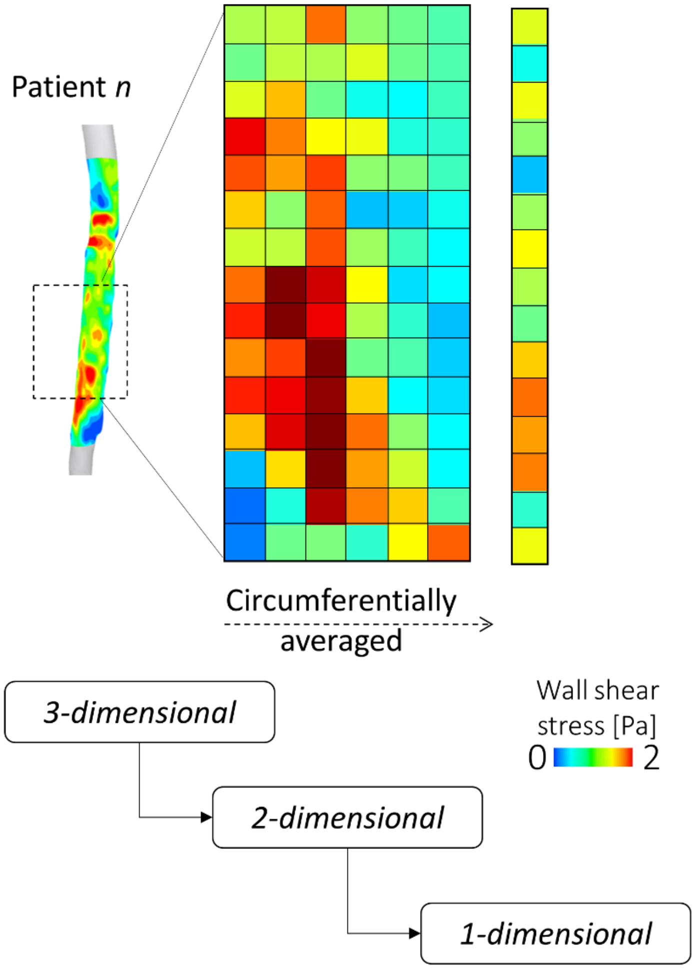

Figure Suppl-2 – Re-organization of the hemodynamic data from three- to one-dimensional maps for matching with one-dimensional morphological data. Explanatory maps of wall shear stress (WSS) of one lesion are here shown. The same procedure was applied to all WSS-based descriptors of all lesions at all follow-ups.

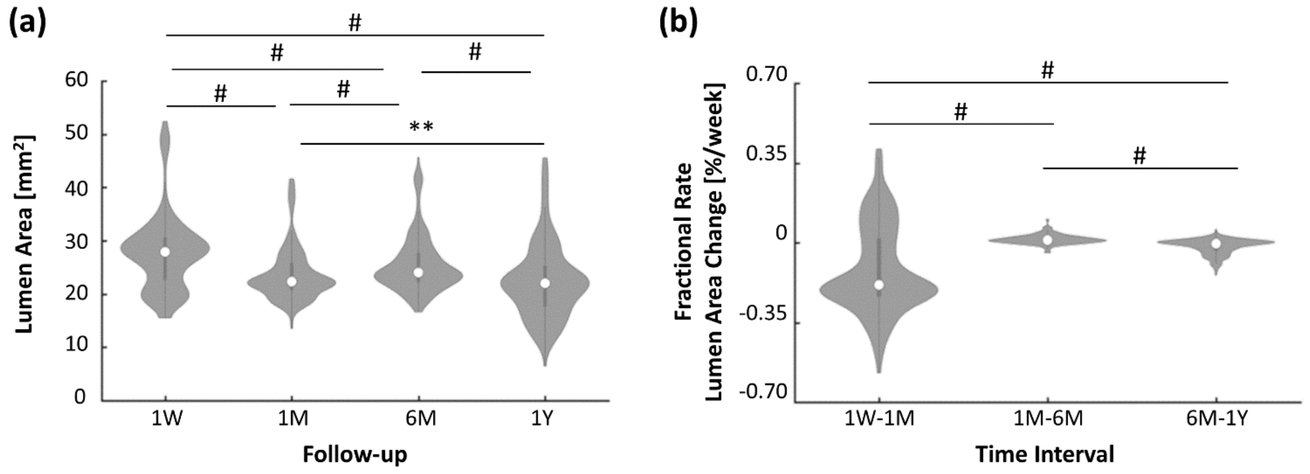

Figure Suppl-3 – (**a**) Distribution of the lumen area of the lesion at different follow-ups (local level of analysis). (**b**) Fractional rate of lumen area change of the lesion computed in the different investigated time intervals (local level of analysis). **1W**: 1 week; **1M**: 1 month; **6M**: 6 months; **1Y**: 1 year; **: $p < 0.01$, **#**: $p < 0.0001$.

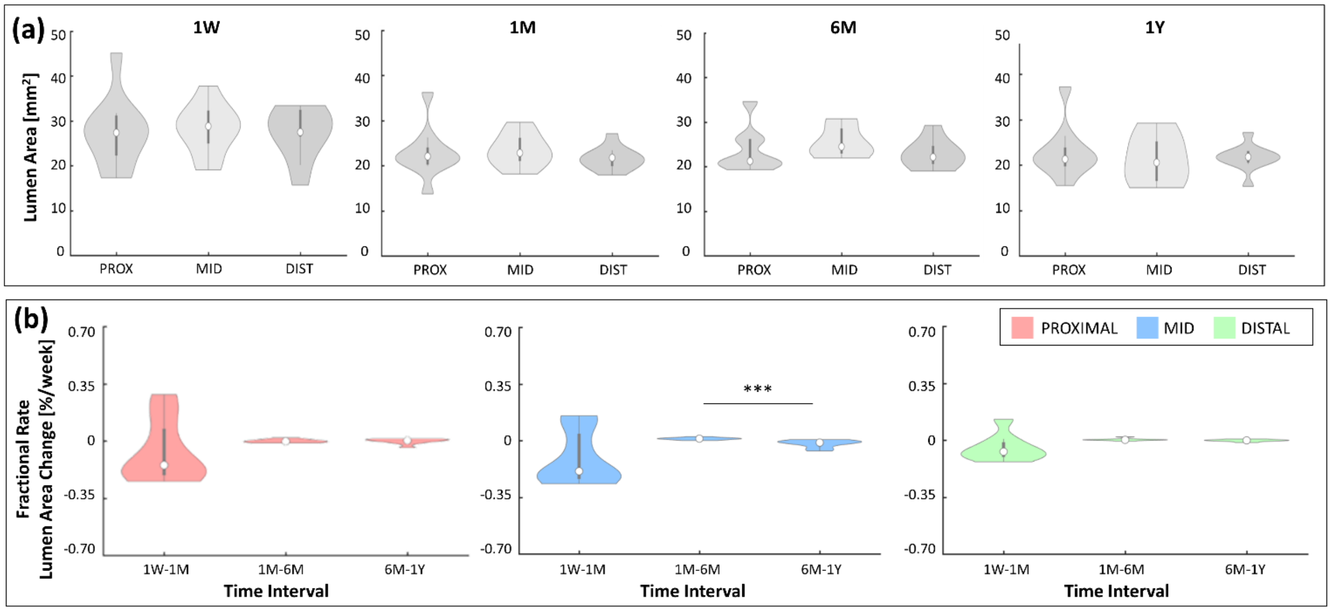

Figure Suppl-4 – Results of the lumen remodeling considering the proximal, mid and distal segments of the SFA lesions (local level of analysis). (**a**) Comparisons of the lumen area distributions at each follow-up. (**b**) Fractional rate of lumen area change in the three investigated time intervals (1W-1M, 1M-6M and 6M-1Y). **1W**: 1 week; **1M**: 1 month; **6M**: 6 months; **1Y**: 1 year; **PROX**: proximal; **DIST**: distal; *: $p < 0.05$; **: $p < 0.01$; ***: $p < 0.001$; #: $p < 0.0001$.

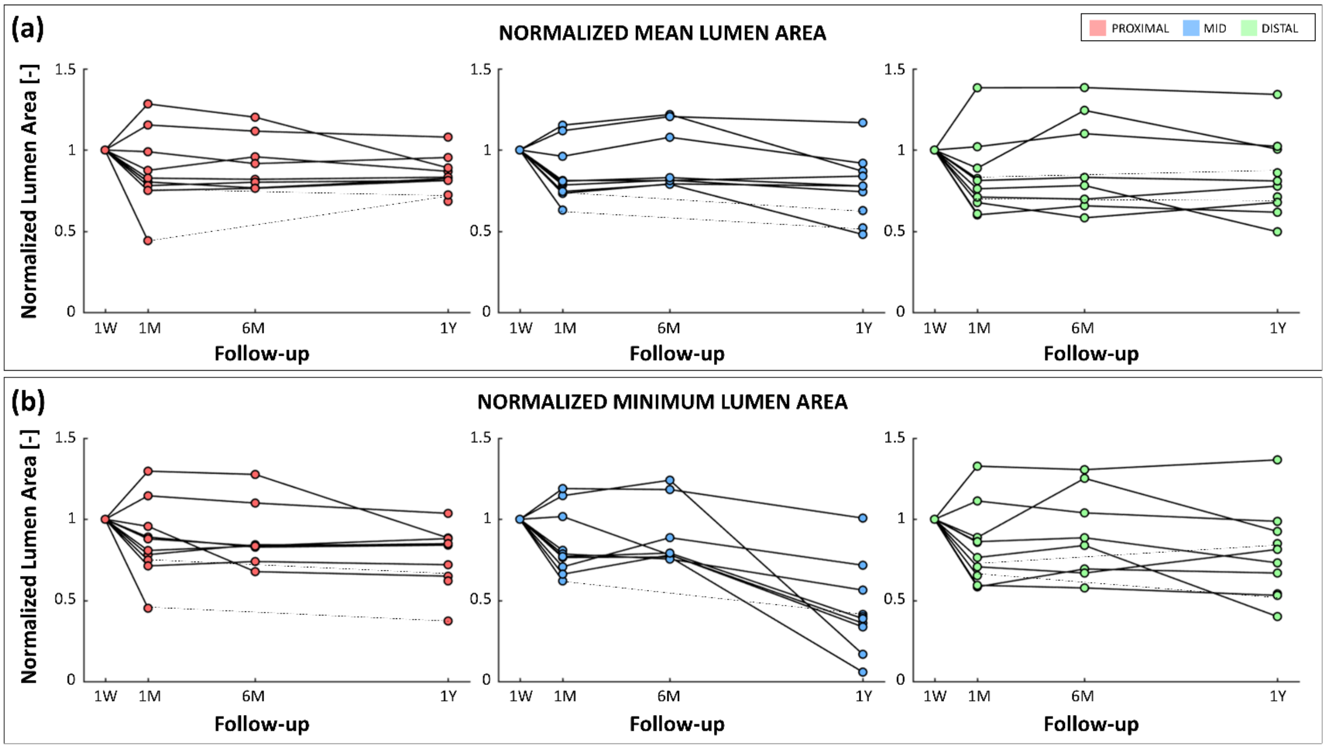

Figure Suppl-5 – Trends over time of the (**a**) mean and minimum (**b**) lumen area, normalized by the initial area at 1W (global level of analysis for proximal, mid and distal segments of the SFA lesions). **1W**: 1 week; **1M**: 1 month; **6M**: 6 months; **1Y**: 1 year.

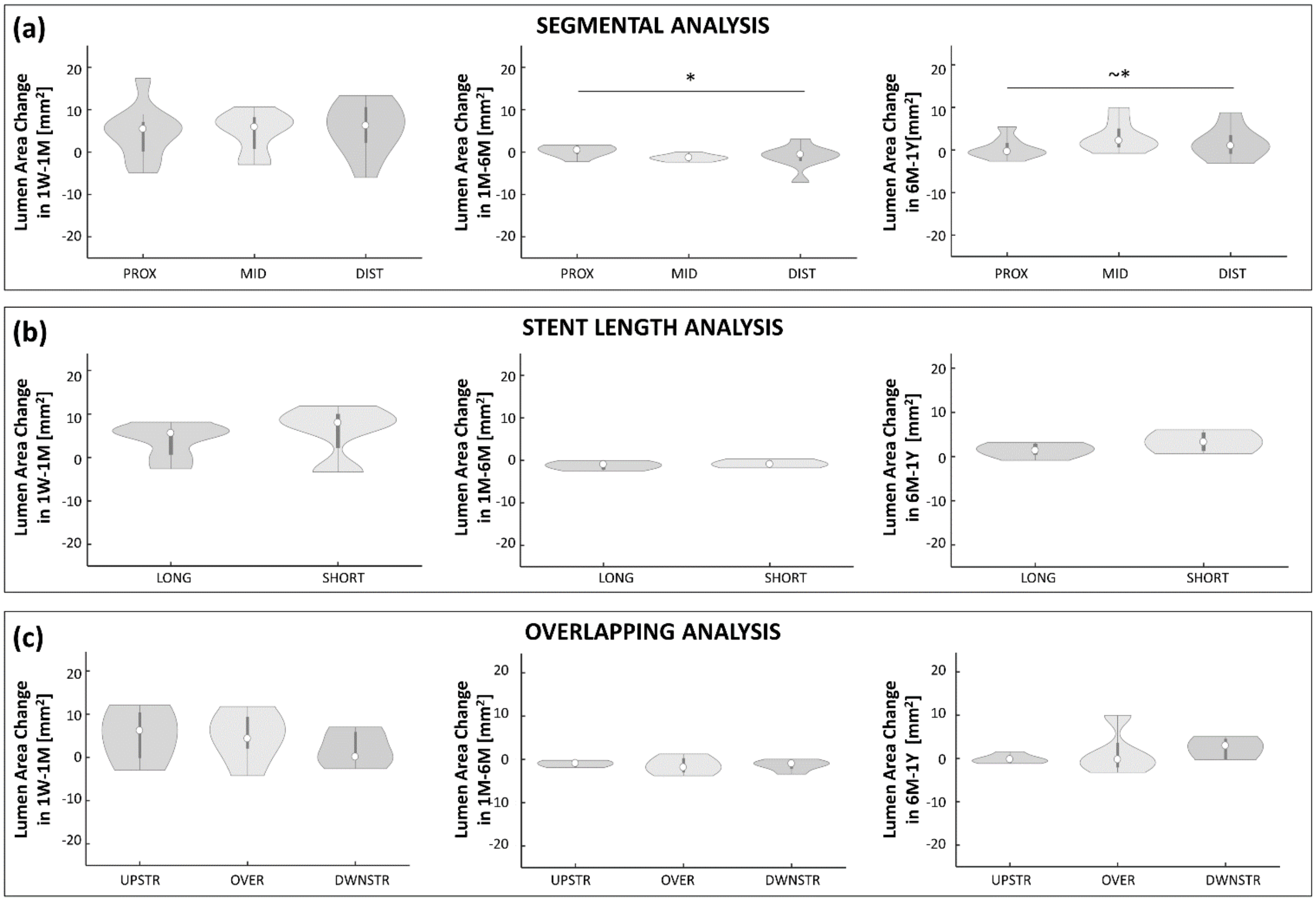

Figure Suppl-6 – Global analysis of the lumen area change distributions in the three time intervals 1W-1M, 1M-6M and 6M-1Y in case of (**a**) segmental analysis: comparison of the lumen area change distributions between the proximal, mid and distal segments; (**b**) analysis of the impact of stent length: comparison of the lumen area change distributions between the long and short device; and (**c**) analysis of the impact of stent overlapping: comparison of lumen area change distributions among the segment upstream from the overlapping, the portion with overlapping, and the segment downstream from the overlapping. **1W**: 1 week; **1M**: 1 month; **6M**: 6 months; **1Y**: 1 year; **PROX**: proximal; **DIST**: distal; **UPSTR**: upstream; **DWNSTR**: downstream; **OVER**: overlapping; ~*: p ~= 0.05; *: $p < 0.05$.

REFERENCES

S-1. Morbiducci, U., D. Gallo, S. Cristofanelli, R. Ponzini, M. A. Deriu, G. Rizzo, and D. A. Steinman. A rational approach to defining principal axes of multidirectional wall shear stress in realistic vascular geometries, with application to the study of the influence of helical flow on wall shear stress directionality in aorta. *J. Biomech.* 48:899–906, 2015.

S-2. De Nisco, G., A. Hoogendoorn, C. Chiastra, D. Gallo, A. M. Kok, U. Morbiducci, and J. J. Wentzel. The impact of helical flow on coronary atherosclerotic plaque development. *Atherosclerosis* 300:39–46, 2020.

S-3. Peiffer, V., S. J. Sherwin, and P. D. Weinberg. Computation in the rabbit aorta of a new metric - the transverse wall shear stress - to quantify the multidirectional character of disturbed blood flow. *J. Biomech.* 46:2651–2658, 2013.

S-4 Peiffer, V., Bharath, A. A., Sherwin, S. J. & Weinberg, P. D. A novel method for quantifying spatial correlations between patterns of atherosclerosis and hemodynamic factors. *J. Biomec*h. *Eng*. 135, 021023, 2013.
